# Solid Ethanol as a Renewable, Low‐Toxicity, Electron‐Beam Direct Write, and Biomedical Material

**DOI:** 10.1002/advs.75341

**Published:** 2026-04-27

**Authors:** Bruno Perdigão, Bingdong Chang, Gwendoline A. E. Anand, Lechan Tao, Kayeon Kim, Anne Zebitz Eriksen, Malte Alexander Schönhoff, Guilherme Ferreira, Xiyuan Liu, Su Genelioglu, Joachim Lyngholm‐Kjærby, Neha Zahoor, Johan Ulrik Lind, Alice Bastos da Silva Fanta, Thomas Willum Hansen, Changsi Cai, Anpan Han

**Affiliations:** ^1^ Department of Civil and Mechanical Engineering Technical University of Denmark Kgs. Lyngby Denmark; ^2^ Department of Chemical Engineering Instituto Superior Técnico Universidade De Lisboa Lisbon Portugal; ^3^ Department of Neuroscience Copenhagen University København N Denmark; ^4^ Department of Health Technology Technical University of Denmark Kgs. Lyngby Denmark; ^5^ National Centre for Nano Fabrication and Characterization Technical University of Denmark Kgs. Lyngby Denmark

**Keywords:** brain machine interface, cross‐linking chemistry, ice lithography, scaffolds, thermoset polymer

## Abstract

3D ice lithography (3DIL) is an emerging direct‐write technique that fabricates intricate 3D structures using frozen precursors. Here, we report the use of ethanol as a renewable and low‐toxicity precursor for 3DIL, intended for the first time for the fabrication of intricate porous microstructures for in vitro and in vivo biomedical applications. The first nanoindentation analysis of 3DIL materials reveals mechanical properties (Young's modulus 2–4 GPa) comparable to biocompatible polymers. TEM shows that the material is an amorphous carbon that undergoes controlled graphitization under annealing at very high temperatures (1300°C). Due to its chemical composition, mechanical properties, and stability in water, cross‐linked ethanol scaffolds support in vitro endothelial cell adhesion and proliferation with high confluency. Patterned neurostimulation electrodes implanted in mouse brains elicit no significant increase in astrocytic or microglial activation, indicating excellent in vivo biocompatibility. Additionally, we present for the first time the use of optically transparent substrates and the first patterning of neurostimulation electrodes using 3DIL. This study positions 3DIL using ethanol as a versatile, direct‐write technique using renewable precursors to produce novel microdevices in biomedical engineering.

## Graphical abstract

3D ice lithography (3DIL) enables the fabrication of intricate submicrometer objects using ethanol as a renewable starting material. This study combines process optimization, structural and material analysis, and biomedical applications, from cell culture scaffolds to the patterning of neurostimulation electrodes, demonstrating performance in both in vitro and in vivo settings.

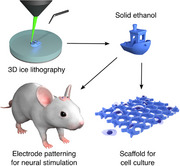

## 1 Introduction

Two‐photon polymerization (2PP) and focused electron beam‐induced deposition (FEBID) are direct‐write (DW) methods that fabricate intricate 3D devices at the micro‐nano scale [1, 2, 3]. In 2PP, the encounter of two photons results in a nonlinear absorption that induces local polymerization and results in sub‐100 nm features [4]. This technology requires photosensitive materials, including acrylic, epoxy, and (in)organic hybrid compounds [5]. 2PP produces complex polymeric devices with optical, mechanical, and life sciences applications [5]. In FEBID, an electron beam dissociates a gasified organic precursor that is adsorbed on the substrate surface to produce features down to the 10‐nm range [6]. This technique possesses a large library of starting materials, including metal‐organics containing carbonyl, halogen, or alkyl compounds [7]. FEBID results in 1D‐3D devices with applications in nano‐optics, nano‐magnetism, and sensing [6].

3D ice lithography (3DIL) is a recent submicrometer DW method that is intrinsically different from and complementary to 2PP and FEBID [8, 9]. Ice lithography (IL) involves vaporizing a precursor, freezing it as an ice layer over the cryogenic substrate surface, and then selectively cross‐linking the frozen material using a focused electron beam [10, 11]. The stage is then heated back up to room temperature. The unexposed ice sublimates while the cross‐linked material remains as a solid network with features as small as 4 nm [12]. In 3DIL, the process is repeated layer‐by‐layer to obtain a 3D object. IL works with an ever‐expanding range of materials, including compounds like water, anisole, or nonane [8, 13] and organometallics [14, 15]. In our previous studies, we demonstrated 3DIL's potential for microfluidics and nanophotonic applications [8] and highlighted its possible use for quantum nanoparticle synthesis [14].

Biomedical applications are expanding and demand the use of increasingly smaller devices, requiring micro‐nanofabrication techniques [16]. 2PP can produce cell‐scaffolds [17] and microneedle arrays [18]. To our knowledge, there are no FEBID studies on biomedical applications. In this study, we present ethanol as a 3DIL material for biomedical applications. Ethanol is an organic, non‐toxic, renewable, and easily accessible compound. We developed ethanol‐based 3DIL processes and investigated the material properties of 3DIL‐processed ethanol ice. Noticeably, we have performed mechanical characterization and in situ heating experiments under transmission electron microscopy (TEM) for the first time. Uniquely, we investigated 3DIL‐processed ethanol 3D objects for both in vitro and in vivo biomedical applications. Not only does it showcase the potential for 3DIL as a DW technique using renewable materials to fabricate complex and fully customizable micro‐scale biocompatible materials, it also offers additional breakthroughs in 3DIL with (i) use of optically transparent substrates (ii) patterning of neurostimulation electrodes, and (iii) insights into the functionalization of 3DIL materials through post‐processing.

## 2 Results and Discussion

**FIGURE 1:**
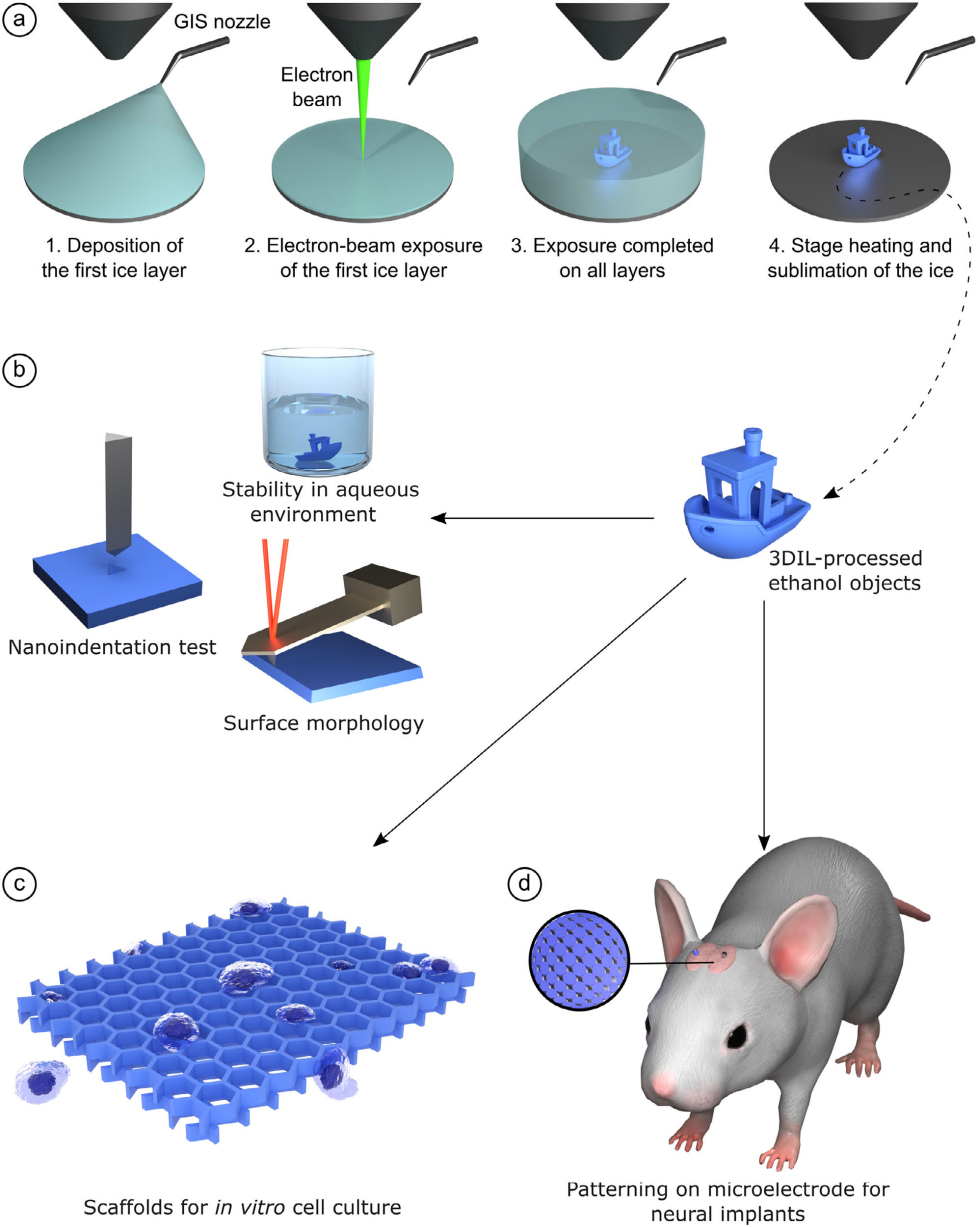
Graphical summary presenting (a) The 3DIL process using ethanol as a starting material. (b) Characterization of 3DIL cross‐linked ethanol, including e.g., stability in water, nanoindentation, and atomic force microscopy. (c) In vitro study with 3DIL cross‐linked ethanol scaffolds for cell culture. (d) In vivo study with implantation of neurostimulation electrodes patterned using 3DIL and ethanol ice.

Figure 1 provides an overview of this paper. We present the 3DIL manufacturing process (Figure 1a), which is facilitated by a custom IL instrument [8, 19]. In step one, the IL gas injection system (GIS) injects gaseous ethanol that freezes over the cold substrate and becomes the first layer. In step two, the electron beam selectively cross‐links the ethanol ice layer. Steps one and two are repeated until the target object is fully cross‐linked, as depicted in step three. In step four, the stage is heated up, and the unexposed ice sublimates, leaving behind the final 3DIL cross‐linked ethanol structure. Then, the 3DIL print produced after optimization of the manufacturing process undergoes material and mechanical studies to investigate its composition and characteristics, as depicted in Figure 1b. Then, we fabricate optimized devices for biomedical applications: scaffolds for in vitro cell culture (Figure 1c) and patterning of neurostimulation electrodes for chronic implantation (Figure 1d).

### 2.1 Model for 3DIL Processing of Ethanol Ice, Process Optimization, and Materials Characterization

We will start by establishing 3DIL processes based on ethanol and introduce important processing parameters to achieve 3D microobjects. Different characterization techniques will then be performed to thoroughly investigate the material properties of 3DIL‐processed ethanol.

**FIGURE 2:**
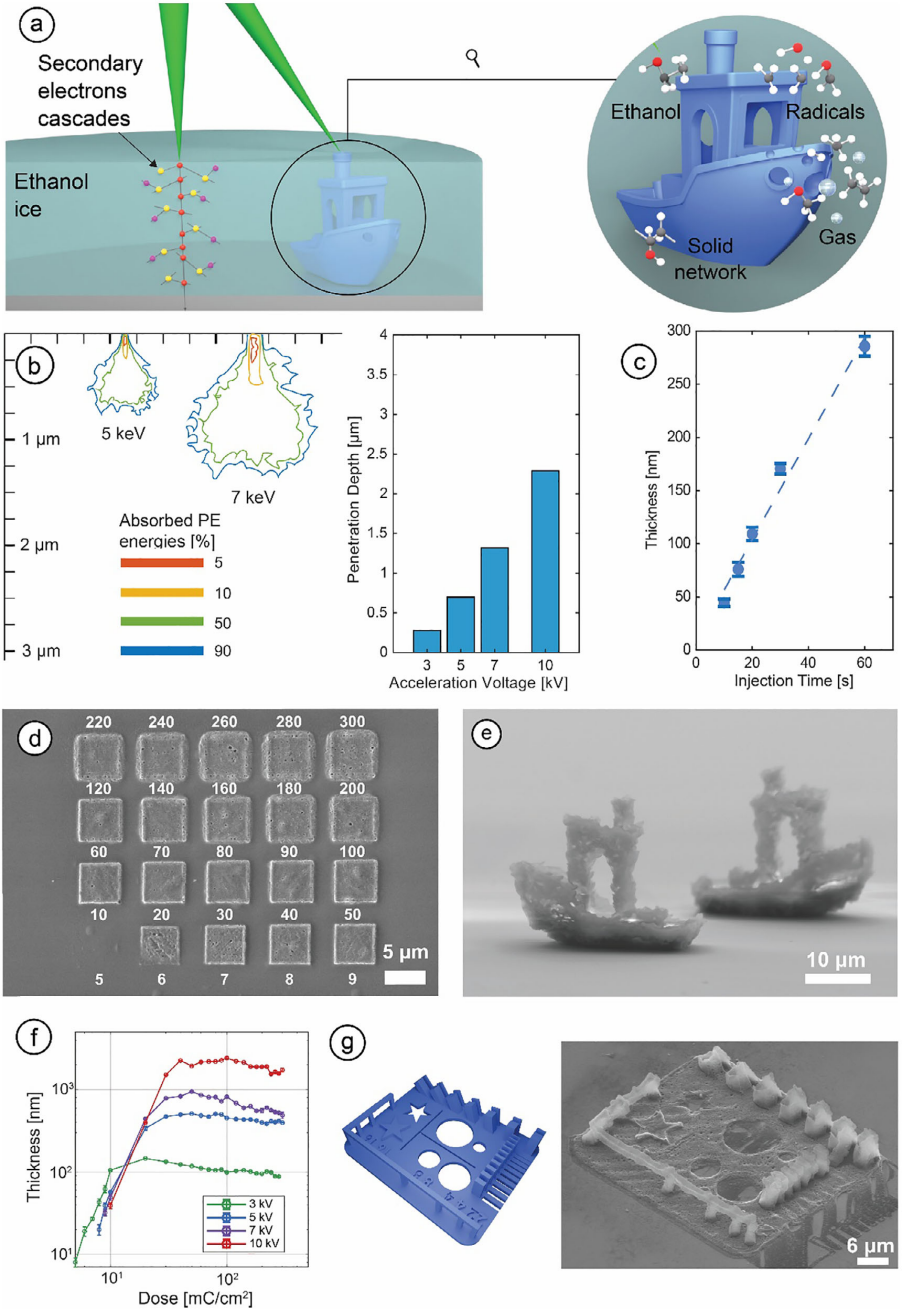
Optimization of the 3DIL process. (a) Theoretical model of the interactions between the electron beam and the ethanol ice. (b) Monte Carlo simulations of the interaction volumes for 5 and 7 kV (on the left) and their penetration depths for 3, 5, 7, and 10 kV (on the right). (c) GIS injection time vs. ice layer thickness at 7 kV. (d) Dose test at 7 kV with the dose in mC/cm^2^. (e) SEM images of 3DBenchy boats fabricated using 5 kV and a dose of 100 mC/cm^2^. (f) Dose curves at 3, 5, 7, and 10 kV. (g) Benchmark test CAD design (on the left) and SEM image of 3DIL print (on the right).

Figure 2a presents our understanding of the 3DIL processing of ethanol ice. On the left, the primary electrons (PE) interact with ethanol ice on a silicon substrate. As the electron beam penetrates the material, it generates cascades of secondary electrons (SE) through inelastic scattering near the PE path. The first cascade of secondary electrons (in red) initiates a second cascade (in yellow), which, in turn, generates a third cascade (in purple). On the right, we hypothesize that the secondary electrons break chemical bonds in the ethanol ice, leading to the formation of radicals. These radicals include e.g., OH*, H*, CH_3_*, CH_2_*, CH_2_OH*. Recombination of radicals results in stable compounds containing C, H, and O, which would remain as a solid network after the stage is heated up. In contrast, gaseous by‐products may include CH_4_, H_2_, (CH_3_)_2_, CH_3_OH, or others, which would result in cavities and pores in the final structure. Our understanding of the molecular‐level interactions is different from FEBID, where radicals generated by electron‐gas interactions drive the deposition of carbonaceous and metallic networks [20, 21].

To optimize the 3DIL process for ethanol, we determine the interaction volumes or voxels, which are the building blocks of 3D printed structures. During the printing process, it is necessary to strategically overlap the voxels horizontally and vertically to guarantee a good adhesion of the layer itself and between adjacent layers [8]. We use Monte Carlo simulations [8] to obtain the voxels at different acceleration voltages (Figure 2b). On the left, the Monte Carlo simulations illustrate the garlic‐shaped interaction volumes of the electron beam in ethanol ice, with a higher acceleration voltage equating to a larger volume. This means that we can print structures at a higher resolution using lower PE energy or achieve high throughput using higher PE energy. On the right, we present a plot of the simulated penetration depth for different acceleration voltages [8]. This is a starting point for how deep we can expect electrons to reach into the ice, and therefore of how thick each layer can be.

We then determined the relationship between the injection time and printed layer thickness (Figure 2c). Injection time refers to the period during which our GIS introduces gas. Because the vaporized ethanol freezes over the sample to become the ice layer, the amount of gas added to the sample is directly proportional to the ice thickness. We quantified the thickness of ice cross‐linked at 7 kV for several injection times. We obtained a linear relation with a rate of 285 nm/min.

After this, we did a parameter optimization study to identify the optimal acceleration voltage and dose to obtain cross‐linked structures that precisely replicate the CAD model. The dose tests consisted of cuboids arranged in a 5 × 5 grid, with each cuboid cross‐linked at a different dose ranging from 5 to 300 mC/cm^2^. Figure 2d is a dose test produced at 7 kV. We printed each dose test at different acceleration voltages. We then measured each of the cuboid thicknesses to obtain dose curves, illustrating the relationship between dose and cross‐linked thickness at different acceleration voltages (Figure 2f). The dose curves all follow a linear increase (log‐log scale) at lower doses for 5, 7, and 10 kV. Then the dose curves reach a plateau after passing the critical dose [14]. Agreeing with the Monte Carlo simulations, a higher acceleration voltage generates a higher layer thickness.

To further validate the 3DIL process, we manufactured 3D objects, such as 3DBenchy boats (Figure 2e), and a benchmark sample (Figure 2g, [8]). For most 3D prints, we selected 5 kV to obtain the optimal trade‐off between a high resolution and a sufficient throughput. For print requiring higher resolution features, such as the benchmark sample, we used 3 kV. 3DBenchy boats show a high shape fidelity with well‐formed overhangs. The porous 3DBenchy agreed well with our model (Figure 2a) that electron‐ethanol ice interactions would produce volatile compounds, which would result in a porous 3D print. The 43‐layer benchmark model tests typical 3D printer abilities and features, including cuboids, fingers, bridges, and overhangs. The cuboids and fingers have a distance or width ranging from 0.2 to 2 µm with an increment of 0.2 µm. The bridges feature distances between pillars varying from 4 to 32 µm, with progressively larger increments. The overhangs are constructed with angles that range from 45° to 90° in 7.5° increments. The test also features engraved lines throughout circular and star‐shaped holes, an extruded star, and the numbers two, four, eight, and sixteen, all of which are engraved and extruded. There is a visible drift between the initial layer and the subsequent ones. The bridge and pillars are printed, but a layer shift is visible due to motion between layers during the fabrication process. The layer shift is caused by thermal drift of our cryostage, and it is compensated for by thermal stabilization [19]. The engraved lines and numbers are not visible, but the extruded shapes are well‐defined.

Unlike gold dimethyl acetylacetonate (AuDA) [14] and nonane [8] 3DIL precursors, the printed ethanol objects exhibit high porosity. This contrast is particularly evident in the 3DBenchy prints, where ethanol‐based structures demonstrate visible porosity, which is absent in counterparts fabricated with AuDA or nonane [8, 14]. This is also true for the benchmark prints. From an energy density perspective, we expected ethanol to exhibit a higher critical dose than AuDA and nonane due to its smaller molecular weight. This was validated in our experiments, as organometallic and alkane precursors require significantly lower critical doses. For example, at 5 kV, the critical dose for AuDA was 10 mC/cm^2^ [14] compared to 100 mC/cm^2^ for ethanol. The same is true for nonane, which requires a lower dose of 12.5 mC/cm^2^ at 5 kV [8]. The benchmark test made using ethanol exhibits a drift between the initial and subsequent layers.

**FIGURE 3:**
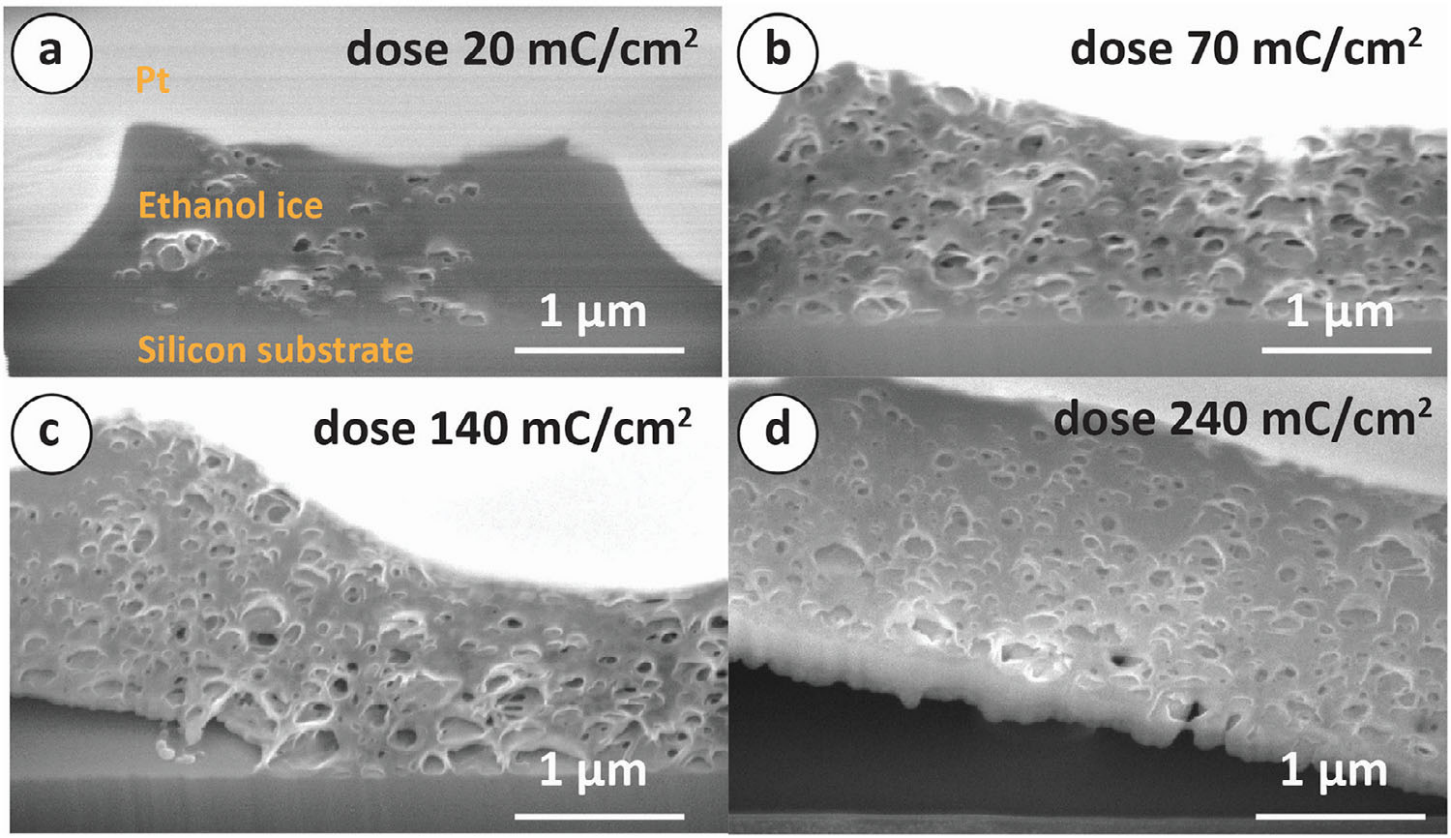
Porosity of printed ethanol ice patterns with different doses: (a) 20 mC/cm^2^. (b) 70 mC/cm^2^. (c) 140 mC/cm^2^ and (d) 240 mC/cm^2^.

It is worth mentioning that 3DIL‐processed ethanol is the first material to yield systematically porous structures. Here, we examined the porosity by sectioning the printed patterns via focused ion beam scanning electron microscopy (FIB‐SEM). A series of dose test patterns, printed with a PE of 10 kV and varying doses, were analyzed. A protective platinum layer was deposited on top of the patterns, after which Ga^+^ ions were used to mill cross‑sections for comparison (Figure 3). It can be clearly seen that a higher dose can generate pores with a higher spatial density. For applied doses of 20, 70, 140, and 240 mC/cm^2^, the density of pores is measured to be 12.2, 17.4, 31.8, and 69.8 µm^−2^ correspondingly. While the pore density increases with dose, there is no clear correlation between the pore size and applied doses, and the pore diameters range between 20 and 200 nm. This inherent porosity motivated our investigation into its potential for in vitro and in vivo biocompatibility. Importantly, the presence of porosity also broadens the application potential of ethanol‐based 3DIL. Fields such as catalysis and biosensing would all benefit from high surface‐to‐volume ratios. By leveraging these properties, ethanol‐based 3DIL could become a versatile platform for fabricating functional micro‐ and nanoscale devices across multiple disciplines.

**FIGURE 4:**
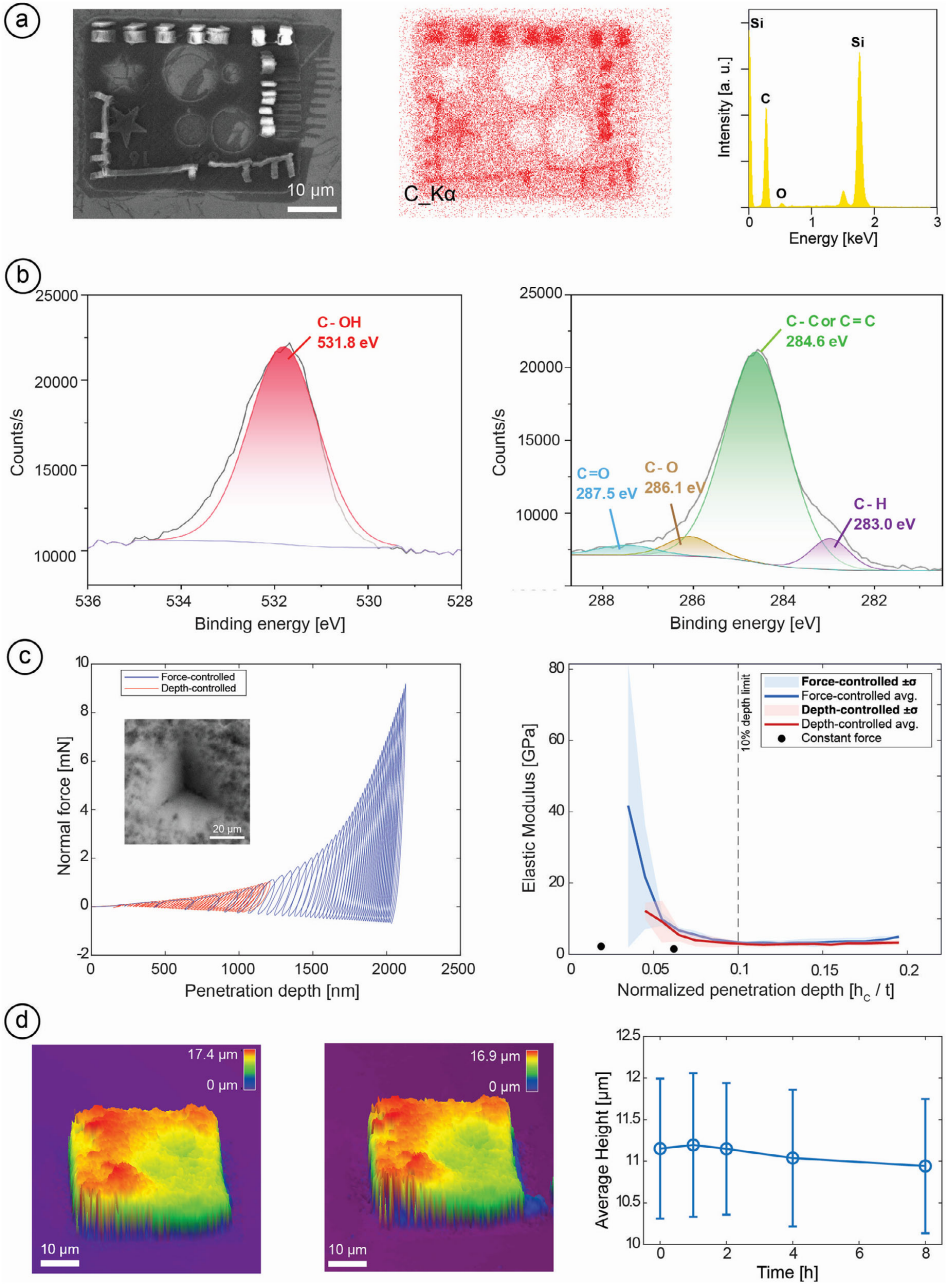
Characterization of 3DIL cross‐linked ethanol. (a) EDS analysis of the benchmark test. (b) XPS analysis with O 1s (left) and C 1s (right) signals. (c) Nanoindentation study with cyclic loading curves and an SEM picture of the hardness indent (left) and the obtained Young Modulus curves for each loading type with the standard deviation (right). (d) Water stability study with the confocal top‐view measurements generated as 3D views of the object as printed (left) and after 8 h (centre), and the average object height with the surface roughness as standard deviation (right).

After optimizing the 3DIL process, we characterize the 3DIL cross‐linked ethanol material. Energy Dispersive X‐ray Spectroscopy (EDS) (Figure 4a) suggests that the material composition is primarily composed of carbon with localized oxygen presence. The EDS spectrum confirms the presence of C and O with peaks at 0.28 keV (C Kα), and at 0.52 keV (O Kα), respectively. Peaks corresponding to silicon (Si) originate from the substrate, and a minor shoulder between 1 and 1.5 keV is attributed to the aluminum sample holder.

To characterize the chemical states of C and O in the cross‐linked ethanol ice, we performed X‐ray photoelectron spectroscopy (XPS) measurements. We analyzed O 1s and C 1s signals specifically (Figure 4b). Since both elements are also present in adventitious carbon and native silicon oxide on sample surfaces, it is important to etch the surface materials away by a monochromatic argon ion sputtering. On the O 1s spectrum, we observed a peak located at 531.8 eV, which fits well with the characteristic peak of the ethoxy group (C_2_H_5_O‐) at 531.7 eV [22]. Since the O 1s signal in unprocessed molecular ethanol is present at 533–535 eV [23], we should assume a high degree of O─H covalent bond breaking in the exposed ethanol ice [22]. We could also see a slight shift of O 1s toward higher binding energy (BE), which could be caused by the existence of a carbonyl group that gives the O 1s signal from 531.8 to 532.2 eV [24, 25]. For the C 1s spectrum, a major peak can be clearly seen at −284.6 eV, which corresponds to the existence of hydrocarbon (C─C or C═C) [26, 27]. The fitting of the C 1s spectrum suggests a shoulder at 286.1 eV and a tail at 287.5 eV, which can be attributed to C─O and C═O, respectively [26, 28]. A secondary peak at a lower BE of 283.0 eV is also observed, which can be assigned to the sp^3^ C─H group [29]. Although XPS studies of solid‐state ethanol exposed by electron beams are limited, radiolysis processes of liquid ethanol have suggested hydrogen abstraction during exposure [30, 31], generating 2, 3‐butanediol and acetaldehyde. These studies agree with our observation of hydrocarbon and carbonyl groups.

To evaluate the mechanical properties of cross‐linked ethanol, we conducted a nanoindentation study (Figure 4c), which is the first time for 3DIL‐processed materials. We used progressive force and penetration depth, in blue and red, respectively, along with two tests at constant depth, shown by black circles. Results from progressive force and penetration depth follow a similar trend with higher initial values at lower penetration depths, followed by a plateau at around 2–4 GPa for shallower penetration depths. The higher initial values are likely due to surface roughness and incomplete contact, as well as potential instrumental artefacts known to affect the accuracy of the initial unloading curves in polymers [32]. The printed structure for nanoindentation is presented in Figure S1, and local porosity can be observed at different nanoindentation sites (Figure S2). The porosity is observed to be uniform across the surface, which gives an average modulus of around 3.03 GPa with a variation of 14%. The statistics of modulus at different indentation sites are presented in Figure S3.

We assessed the structural stability of 3DIL cross‐linked ethanol under aqueous conditions (Figure 4d) to determine its inertness in potential biological applications. We manufactured a flat‐top structure held by a smaller‐width central pillar. This structure permits the solid network to expand or contract freely, allowing us to obtain potential swelling information. We used a confocal microscope to take top‐view measurements of the structure as‐printed and after being in deionized (DI) water at 40°C (relevant for physiological conditions) for 1, 2, 4, and 8 h. The results show that the structure displays structural stability with no clear trend in contraction or expansion, and the highest deviation is less than 2% compared with the original object. The standard deviation is represented by the surface roughness, *S_a_*, which remained the same throughout the experiment. These results suggested that 3DIL cross‐linked ethanol bonds are, as expected, highly cross‐linked and therefore inert in water.

These findings give us deeper insights into the 3DIL cross‐linked ethanol and its characteristics. As ethanol is constituted of C, O, and H, we expected the EDS analysis of cross‐linked ethanol to result in a high concentration of C and traces of O, which is the case here. Notably, the results are similar for C as they are when using AuDA as the precursor [14]. This could indicate that 3DIL materials tend to be a carbon matrix with traces of other elements or embedded nanoparticles [14]. The XPS further strengthens this analysis by revealing similar findings to a previous investigation on as‐printed thin films made using AuDA precursor: (i) both show C 1s peaks around 284.6–284.7 eV, typical of C─C and C═C bonds. (ii) The cross‐linked ethanol shows a shoulder at 286.1 eV and a tail at 287.5 eV corresponding to C─O and C═O groups, while the carbon‐gold material displays a similar oxidized carbon “tail,” though not as resolved, and (iii) The O 1s spectrum of the ethanol‐based sample includes a peak at 531.8 eV, consistent with ethoxy groups, whereas oxygen states in the carbon‐gold sample are inferred from the C 1s profile [14]. The nanoindentation offers insights into the biocompatibility of 3DIL cross‐linked ethanol. This is because biocompatible materials should have similar mechanical and chemical properties when compared with the biological environment [33]. Brain tissues, for instance, have a Young's modulus of around 1 kPa, while typical brain‐machine interface materials have a Young modulus reaching up to 100 GPa and more [33, 34, 35, 36]. Our analysis shows that 3DIL cross‐linked ethanol has a Young's modulus between 2–4 GPa using progressive force or penetration depth, and between 1.5 and 2.25 GPa for constant shallow penetration depths, which is similar to polyvinyl alcohol (PVA)’s Young's modulus of 2.75 GPa [37]. This further strengthens the claim that 3DIL can use renewable materials, i.e., ethanol, to produce biocompatible devices. Our investigation shows cross‐linked ethanol is inert in aqueous conditions and at the body's basal temperature. This demonstrates a high structural and chemical stability, which is sought out in many biomedical applications, including but not limited to permanent implants and scaffolds for cell culture [35, 38, 39]. The exploration of biomedical applications using 3DIL is in its infancy. Two more studies focused on patterning biological or living matter using 3DIL [40, 41]. Previous work also demonstrated microfluidics applications [8]. This emerging technology proves to be a new tool able to make new devices using starting materials that 2PP or FEBID are, to the best of our knowledge, not yet able to employ.

**FIGURE 5:**
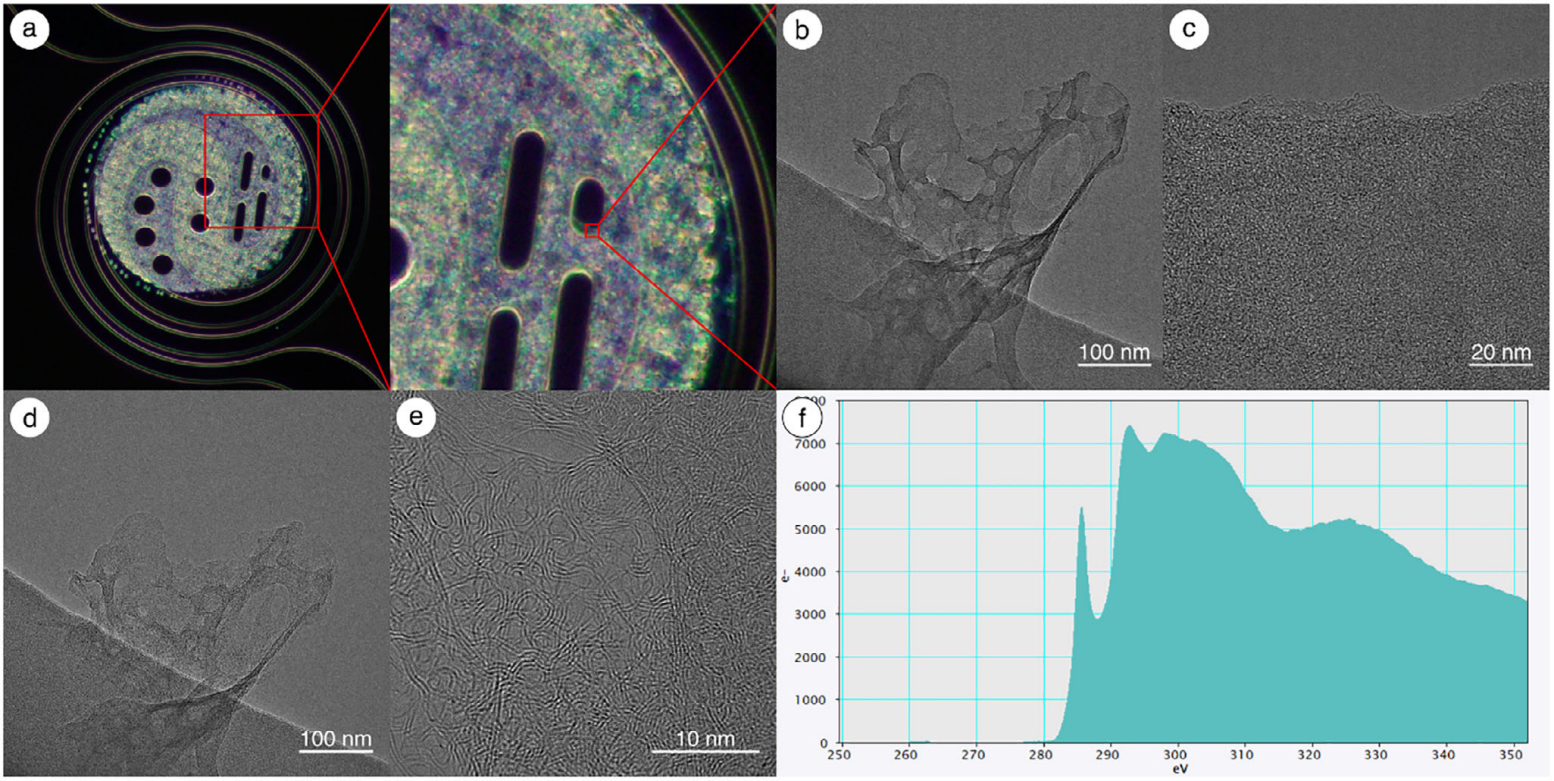
High temperature annealing of cross‐linked ethanol. (a) LOM picture of the TEM heating platform where we printed a 3DIL disc with a grid on top. (b) Low magnification image acquired at 100°C. (c) Initial graphitization was observed at the edge of the cross‐linked ethanol structure when reaching 1300°C. (d) Same area as b) after 5 h at 1300°C. (e) Graphitization of the cross‐linked ethanol observed after 6 h at 1300°C. (f) EELS spectrum showing the carbon K‐edge.

We conducted TEM measurements to follow the structural evolution of our 3DIL cross‐linked ethanol during temperature ramping. We expected the 3DIL print to exhibit little to no deformation due to our previous findings highlighting the thermoset nature of 3DIL organometallic gold [14]. Figure 5a shows the TEM heating platform with an emphasis on a piece of cross‐linked ethanol attached to the edge of the Si_3_N_4_ window. The TEM images agreed with the SEM images that the cross‐linked ethanol is much more porous than other IL‐processed materials, such as alkanes, aromatics, and organometallic precursors [8, 14, 42]. Figure 5b presents the 3DIL print at 100°C. Below 100°C, the cross‐linked ethanol is amorphous, as diffraction analysis does not reveal any crystalline arrangement. However, it also depicts initial graphitization at the top edge (Figure 5c). Figure 5d shows cross‐linked ethanol after 5 h of annealing at 1300°C. The carbonaceous material exhibits a shape reduction of less than 10%, indicating excellent dimensional stability even at extremely high temperatures. This observation is consistent with our previous findings that 3DIL cross‐linked materials behave as thermosets, retaining their structure under thermal stress due to their cross‐linked networks. Figure 5e shows that the 3DIL processed ethanol undergoes complete graphitization after 6 h at 1300°C with a lattice fringe pattern, typical of graphitization [43]. Moreover, in Figure 5f, we provide the electron energy‐loss spectroscopy (EELS) spectrum showing the carbon K‐edge with distinct π* (−285 eV) and σ* (∼295–310 eV) features, consistent with sp^2^‐hybridized carbon in a graphitized structure [44]. The amorphous carbon nature of 3DIL cross‐linked ethanol is consistent with our previous findings, where we studied 3DIL films produced using organometallic gold [14]. In contrast, our previous study using gold organometallics focused on Au nanoparticle formation at lower annealing temperatures (450°C), which did not promote graphitization [14]. Moreover, the graphitization of 3DIL carbonaceous polymers resembles similar studies conducted on polymeric thin films produced using 2PP and carbon‐based materials [43, 45, 46]. The 2PP thin‐films produced using an IP‐Dip photoresist were also printed on top of a TEM heating chip and annealed in a TEM at 1300°C for several hours, similarly, showing an increasing level of graphitization over time [43]. For the other carbon‐based materials, the graphitization took place ex‐situ and was later observed in a TEM, confirmed by micrographs exhibiting the typical lattice fringe pattern and EELS spectrum with distinct π* and σ* peaks [45, 46].

Our results offer new insights into 3DIL and its ability to produce thermoset materials with high structural stability using renewable precursors. This capability not only highlights the sustainability potential of 3DIL but also opens new opportunities for integrating graphitic carbon into biomedical platforms. Graphite has gained traction in biomedical applications as it can improve hemocompatibility, antithrombosis, and corrosion resistance of, for instance, metallic biomedical implants [47, 48]. Compared to conventional coating or lithographic methods, 3DIL offers a DW strategy for fabricating intricate, high‐resolution 3D structures that retain their geometry pre‐ and post‐graphitization, making it a compelling approach for next‐generation biointerfaces.

### 2.2 3DIL Cross‐Linked Ethanol Scaffolds Support Endothelial Cell Growth In Vitro

**FIGURE 6:**
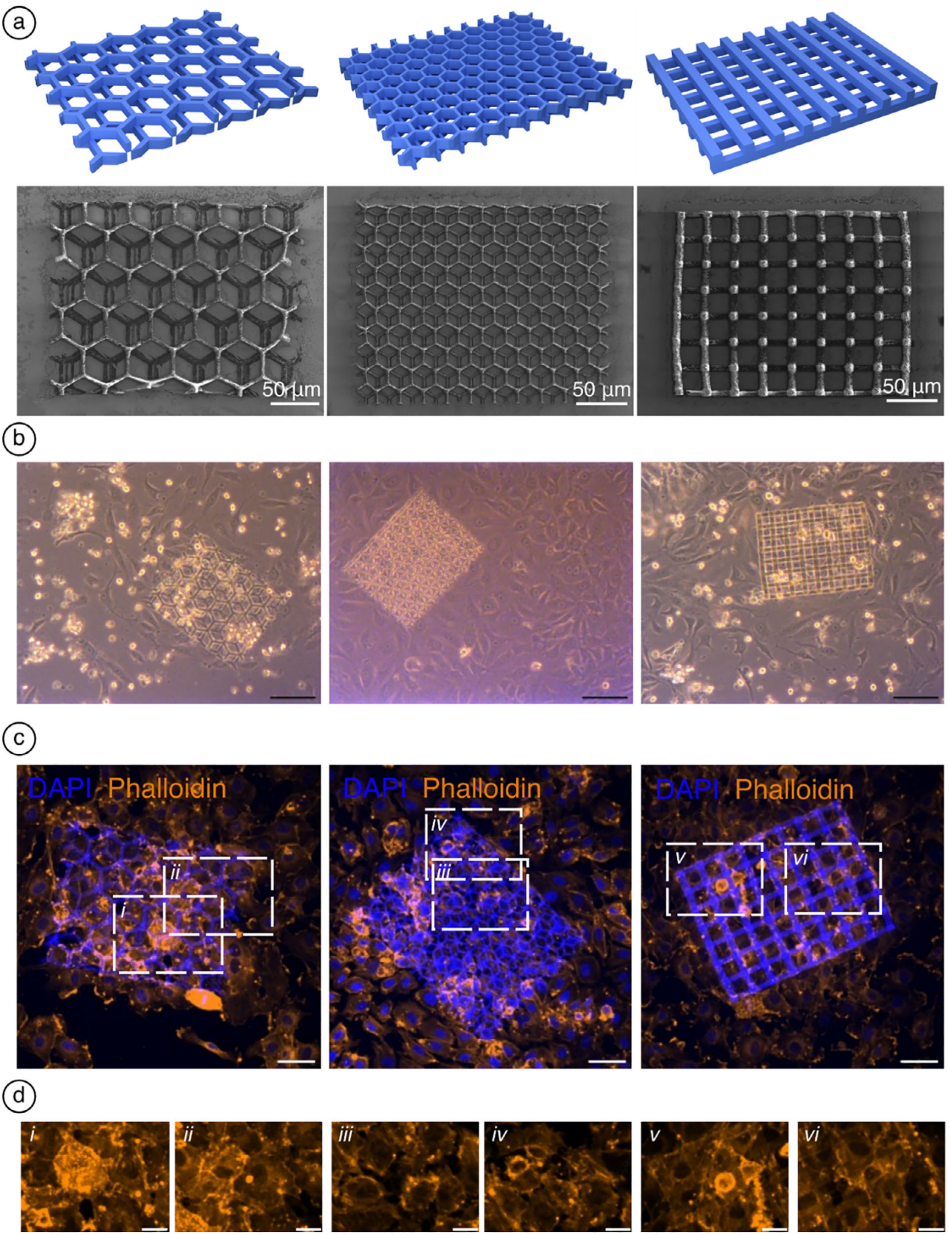
In vitro study of HUVECs on 3DIL‐fabricated scaffolds. (a) CAD designs and SEM images of scaffolds. Left: honeycomb pattern with 40 µm pore size; middle: honeycomb with 20 µm pore size; right: log pile with 20 µm pore size. (b) Phase contrast images of HUVECs on 3DIL‐fabricated scaffolds. Scale bar = 50 µm. (c) Fluorescent micrographs of HUVECs on 3DIL scaffolds. Nuclei stained with DAPI (blue), and cytoskeleton stained with Phalloidin 555 (orange). Scale bar = 50 µm. (d) Zoom‐ins of the phalloidin channel, showing cytoskeleton alignment with the scaffold. Scale bars = 20 µm.

Having demonstrated that 3DIL‐processed ethanol ice is stable in aqueous environments at a range of temperatures, we next explored its potential within biomedical applications. Initially, to evaluate its compatibility with mammalian cell culture, we printed various 3D micro‐scaffolds and cultured human umbilical vein endothelial cells (HUVECs) onto these. Specifically, we printed scaffolds with 20 µm squares, 20 µm hexagons, or 40 µm hexagons (Figure 6a). The scaffold designs consisted of two stacks of hexagonal layers that were purposefully staggered, inspired by previous studies based on 2PP [49, 50]. The scaffolds were printed on optically transparent. conducting, indium‐tin oxide (ITO)‐coated coverslips (Corning glass), to allow for evaluation of cellular morphologies using fluorescence microscopy. All samples were fully confluent after approximately 6 days (Figure 6b), Figure S4. To further evaluate cell adhesion onto the scaffolds, we chemically stained the nuclei and actin filaments of the HUVECs (Figure 6c). The scaffolds exhibited strong auto‐fluorescence in the blue (405 nm) channel, enabling the visualization of cellular organization in response to scaffold geometry with ease. Independent of scaffold geometry, we observed that actin filaments of the cells aligned with the scaffold structure, indicating cell adhesion and spreading onto the support (Figure 6d). We observed no striking differences in cell coverage for the three different scaffolds, independent of their porosity. Similarly, we only observed minimal migration into the scaffold, consistent with the propensity of endothelial cells for forming monolayers. Thus, the 3DIL scaffolds are compatible with in vitro cell culture and appear promising as tunable miniaturized membranes for barrier tissue models and pharmacokinetic transport studies.

These findings provide strong evidence that 3DIL cross‐linked ethanol is biocompatible and suitable for supporting endothelial cell growth in vitro. The alignment of actin filaments with scaffold geometry further indicates that the material enables effective topographical guidance, a desirable feature in tissue engineering scaffolds [51, 52]. Notably, the intrinsic autofluorescence of the cross‐linked ethanol in the 405 nm channel, while potentially limiting for blue‐channel nuclear stains, allows clear visualization of cellular organization, suggesting possible applications in label‐free imaging. Compared to established techniques like 2PP, 3DIL ethanol offers a distinct advantage because it is a low‐toxicity and renewable material, thereby avoiding the risk of subjecting the user to toxic compounds. While 2PP remains attractive for its throughput, 3DIL introduces a complementary strategy that supports material flexibility and can leverage the use of novel and renewable precursors. These results mark the first demonstration of 3DIL's viability for in vitro biomedical applications, specifically as a platform for fabricating miniaturized scaffolds compatible with endothelial culture. This paves the way for further exploration of 3DIL in biomedical applications, offering both a novel fabrication method and access to a previously untapped class of renewable, biocompatible materials for barrier tissue models.

### 2.3 3DIL‐Processed Ethanol Implanted in the Mouse Brain Did Not Increase Astrocytic or Microglial Activity Compared to Control

**FIGURE 7:**
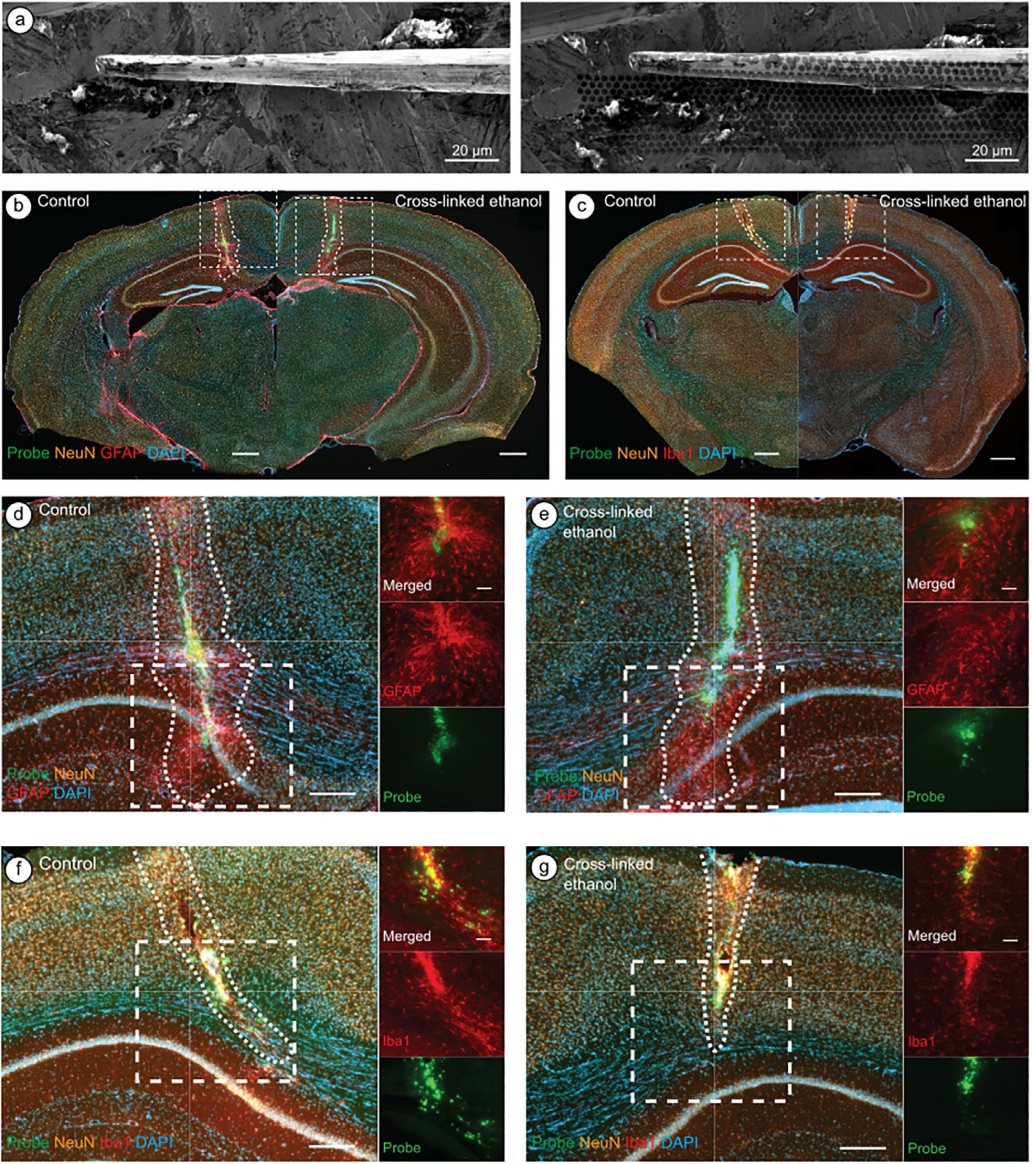
Biocompatibility of cross‐linked ethanol‐coated probes in the mouse brain. (a) SEM images of the pristine (left) and patterned (right) electrode tip. (b) Immunohistochemistry of NeuN (neurons, orange), GFAP (astrocytes, red), and DAPI (blue) in brain hemispheres implanted with control (left panel) and cross‐linked ethanol‐coated (right panel) probes (green). Scale bar: 500 µm. (c) Immunohistochemistry from a separate mouse showing NeuN (neurons, orange), Iba1 (microglia, red), and DAPI (blue), using the same convention as in (b). Scale bar: 500 µm. (d,e) Magnified views from (b) highlighting NeuN (orange), GFAP (red), and DAPI (blue) in hemispheres with control (d) and cross‐linked ethanol‐coated (e) probes (green). Scale bar: 200 µm (left panel) and 50 µm (right panel). (f,g) Magnified views from (c) highlighting NeuN (orange), Iba1 (red), and DAPI (blue) in hemispheres with control (f) and cross‐linked ethanol‐coated (g) probes (green). Scale bar: 200 µm (left panel) and 50 µm (right panel).

**FIGURE 8:**
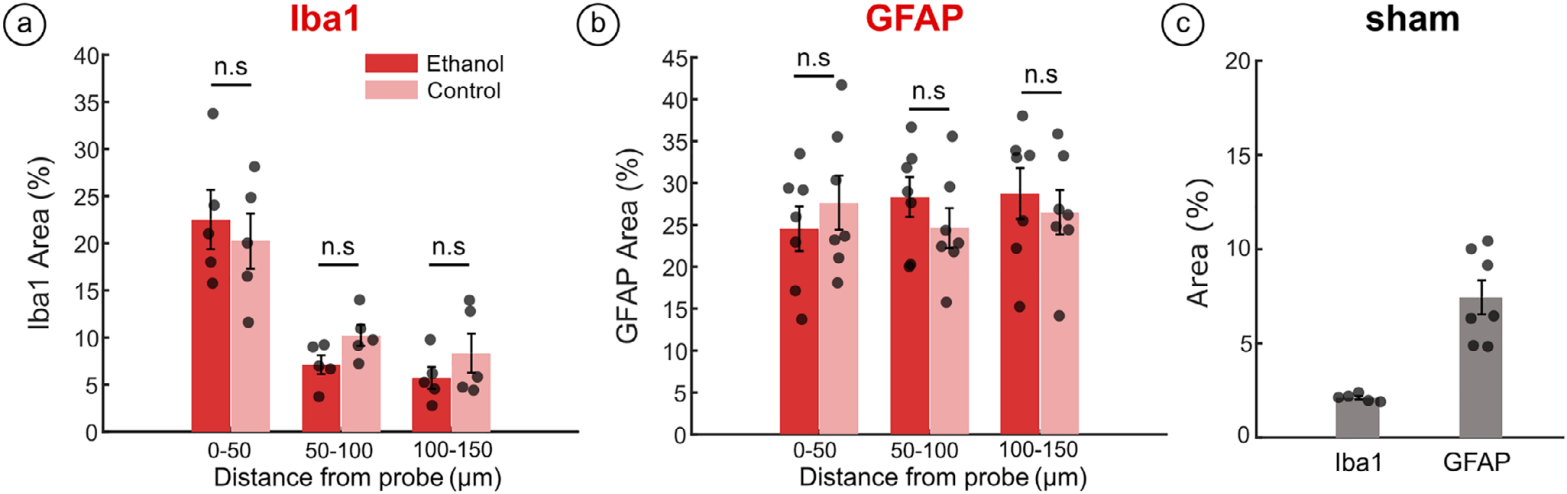
Comparison between cross‐linked ethanol‐coated probes and control probes (a) Bar plots comparing Iba1 signal surrounding the probe tract within the assessed distance for cross‐linked ethanol‐coated probes (red) and control probes (pink). (b). Same as (a) but for the GFAP signal. (c). Sham control group. Dots represent individual regions of interest (ROI); *n* = 4 mice, paired *t*‐test; n.s., *p* > 0.05. See Methods for details.

As we have validated the potential of 3DIL‐processed ethanol for in vitro biomedical studies, we study in vivo applications in this section. Specifically, we investigated the in vivo biocompatibility of cross‐linked ethanol in the mouse brain. We used 3DIL to pattern ethanol ice with neurostimulation electrodes as the supporting substrate (Figure 7a). To evaluate the neuroinflammatory response of cross‐linked ethanol, we assessed astrocyte (Figure 7b, GFAP) and microglia (Figure 7c, Iba1) activation after 14 days following the implantation of probes with and without cross‐linked ethanol using immunohistochemistry (IHC). Statistics have been performed to investigate the glial reactivity with different distances from the probe (Figure S5), and no significant differences in glial activation or glial scar formation were observed between the hemispheres implanted with control and probes with 3DIL cross‐linked ethanol patterns (Figures 7d–g and 8a,b, *p* > 0.05). Meanwhile, sham control analysis (Figure 8c) showed a lower % area occupied by glial cells. These results demonstrate that, at a sub‐chronic time point (>14 days post‐implantation), cross‐linked ethanol does not exacerbate neuroinflammatory responses compared to Pt and parylene C, which are widely used for chronic neural implants in both clinical and preclinical settings [53, 54].

These findings extend the biocompatibility of 3DIL cross‐linked ethanol from in vitro to in vivo contexts. The absence of significant differences in astrocytic (GFAP) and microglial (Iba1) activation between control and cross‐linked ethanol‐coated probes indicates that this material falls within the established biocompatibility range for brain implants, supporting for safe neural interfacing [55]. The use of 3DIL to directly pattern neurostimulation electrodes on probe tips demonstrates the feasibility of integrating this technique into microscale implantable systems. While 2PP can pattern on optical fibre for the optogenetic neurostimulation method (Pisanello et al., 2014), 3DIL complements 2PP by patterning on metals that might reflect light and interfere with the 2PP process.

Given its capacity for high‐resolution, customisable patterning, compatibility with biologically benign precursors, and Young's modulus smaller than normal metallic materials, 3DIL‐processed ethanol holds strong potential for fabricating minimally invasive neural interfaces using renewable precursors. Furthermore, it suggests promise for broader biomedical applications, including long‐term brain–machine interfaces [33, 56, 57, 58], localised drug delivery platforms such as microneedle arrays [59], and other implantable microdevices requiring precise material control and low immunogenicity.

### 2.4 Hypothesis for Solid Ethanol Chemical Transformation Process by 3DIL and Thermal Annealing

**FIGURE 9:**
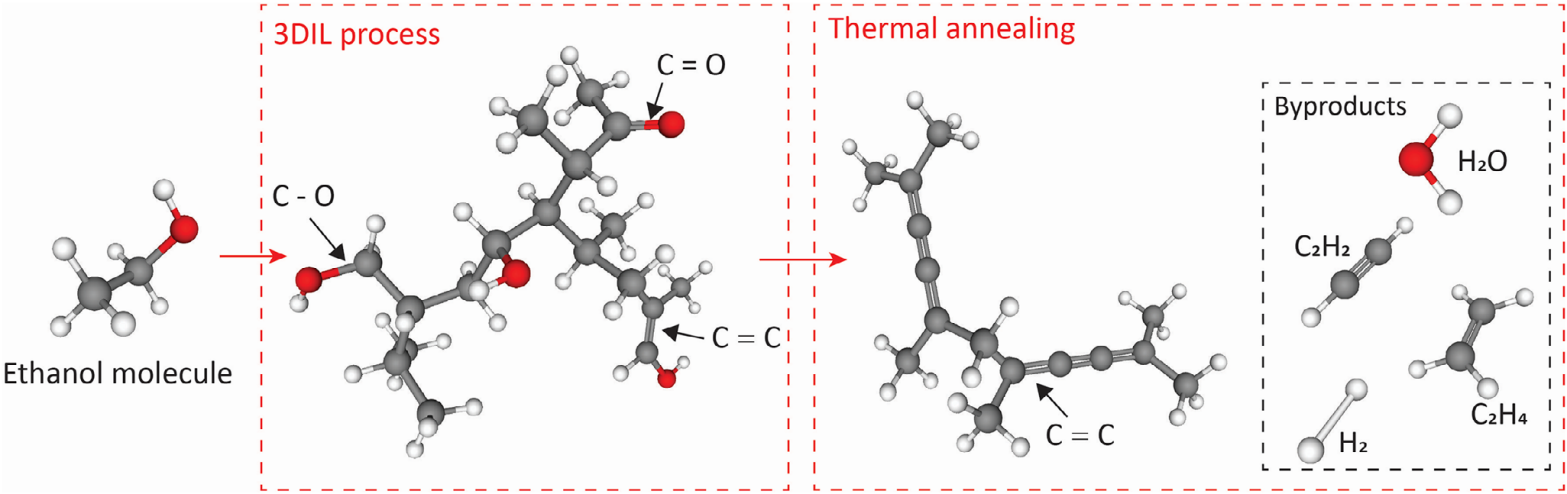
Hypothesized chemical transformation of ethanol molecules by 3DIL and thermal annealing processes.

Based on our material characterization studies (XPS, FIB‐SEM, and TEM), we propose the chemical transformation of ethanol to a cross‐link polymer, which is illustrated in Figure 9. When exposed by electron beams, dehydrogenation and dehydration will happen in ethanol ice, generating hydrocarbons, C═ O, and C─O bonds. C─C and C═C bonds serve as the backbone of the crosslinked ethanol, which is stable at room and elevated temperatures. Since ethanol contains more hydrogen and oxygen than nonane and anisole, more dehydration and dehydrogenation processes are expected during electron‐beam processing, which likely explains the higher porosity. During thermal annealing of cross‐linked ethanol, we observed sp^2^‐carbon formation, consistent with our previous studies on annealing of polymers [45]. We also observed material loss, which is likely due to the generation of byproducts like water, hydrogen, ethene, and acetylene, which are found in earlier studies of ethanol pyrolysis [60, 61].

## 3 Conclusions

In this study, we establish ethanol as a renewable precursor for 3DIL, enabling the DW fabrication of complex microstructures with demonstrated relevance for biomedical applications. We optimised the 3DIL process using Monte Carlo simulations and dose‐response studies to define key parameters for voxel geometry and printing throughput. Notably, we report the first IL patterning on optically transparent substrates, also for the first time, direct patterning on neurostimulation electrodes suitable for in vivo implantation. We further conducted the first nanoindentation study of a 3DIL‐fabricated material, revealing a Young's modulus of 2–4 GPa, consistent with known biocompatible polymers such as PVA. Additionally, we demonstrate that cross‐linked ethanol undergoes controlled graphitization at very high temperatures (up to 1300°C) while preserving structural integrity. We demonstrated biomedical applications both for in vitro and in vivo purposes. We present, for the first time, scaffolds made from cross‐linked ethanol that are fully compatible with in vitro cell culture and offer a promising way to produce customizable miniaturized membranes for barrier tissue models, relevant for pharmacokinetic transport studies. For in vivo applications, we patterned neurostimulation electrodes with cross‐linked ethanol, which elicited incomparable glial activation relative to conventional implants, reinforcing the material's biocompatibility and non‐toxicity.

These findings highlight 3DIL as a competitive DW technology complementary to 2PP and FEBID, especially regarding (i) writing speed to generate sufficient volume of scaffolds and patterns for biological applications. (ii) a solvent‐free and non‐toxic process for biocompatible devices. (iii) a broad range of precursors that are renewable for process sustainability. (iv) flexibility to create 3D objects on various substrates like glasses and microelectrodes. With these attractive advantages, we expect promising applications to be demonstrated in drug delivery, biosensing, and neural interface technologies.

## 4 Experimental Section

### 4.1 3DIL Processing of Ethanol Ice

We used absolute ethanol from VWR Chemicals. The 3DIL process occurs in a custom ice lithography system [8, 19]. We simulated the interaction volume between the electron beam and the ethanol ice using Monte Carlo simulations performed on CASINO v2.5.1.0. The substrate material is set as ethanol with a density of 0.5257 g/cm^3^ in the simulation setup, and 20000 electrons were used in trajectory simulations with a beam radius of 10 nm. We downloaded the 3DBenchy model from Benchy.com and produced our other computer‐aided designs (CAD) models using Solidworks 2023 and Onshape. We sliced the stereolithography (STL) files using Ultimaker Cura 5.9.0. Before and during printing, a liquid nitrogen (LN_2_) tank connected to the cryogenic stage cools down the substrates. The heating process after 3DIL is performed by two steps: first, compressed air is blown through the LN2 tank to raise the cryostage temperature rapidly from −200°C to −20°C within 15 min, afterward a cartridge heater is activated to increase the cryostage temperature further to 30°C in 5 min. This procedure ensures a fast process and effective sublimation of non‐crosslinked ethanol ice. For atmosphere control, the sample was typically loaded in the SEM system and pumped for a few hours to reach a good vacuum level of around 1 × 10^−4^ mbar, before the 3DIL process was started. A good vacuum condition was crucial to achieve a good focus of electron beams and, therefore, the resolution limit of printed structures. The substrates were pristine silicon chips and glass coverslips coated with indium tin oxide (ITO) by Diamond Coatings. The samples were handled by tweezers to avoid contamination on the sample and cryostage, and the position was adjusted manually to have the printed area located close to the nozzle for a uniform coating of ethanol precursors.

### 4.2 Materials Characterization

We used a Gemini Supra from Zeiss for SEM imaging and EDS measurements. We acquired pictures and 3D measurements of our structures using a confocal microscope, LEXT OLS5100 by Olympus. However, because thin cross‐linked ethanol was translucent, we first coated the dose test samples with 10 nm Al at a deposition rate of 1 Å/s using a thermal evaporator by Kurt J. Lesker Company. We then validated the results obtained using confocal microscopy with measurements taken using an atomic force microscope (AFM), Dimension Edge by Bruker.

We conducted XPS measurements using XPS Nexsa by Thermo Fisher Scientific Inc., equipped with monochromatic Al K‐alpha photons at 1486.6 eV. The samples were three structures printed at 5 kV with a dose of 70 mC/cm^2^. The pass energy was 10 eV for all measurements. The pressure of the analysis chamber was around 12.5 × 10^−8^ mbar, the step size of the spectrum was 0.1 eV, and the dwell time was 50 ms. To remove the C and O signal from surface materials (adventitious carbon and native silicon oxide), we used monochromatic argon ion sputtering for 20 s. We processed the XPS data using the Avantage data system by Thermo Fisher Scientific Inc., with a Shirley background type to fit the peaks on the spectra with Gaussian functions.

We tested the stability of the cross‐linked ethanol in deionised (DI) water by Sigma–Aldrich. We used a hotplate for these experiments to keep the sample in DI water at 40°C. We checked the specimen's structural stability over several time points (1, 2, 4, and 8 h) using a confocal microscope, LEXT OLS5100 by Olympus. The height measurement was the average of each corner and the centre, with the surface roughness, *S_a_*, serving as the standard deviation.

We employed a Nanoindentation Tester NHT^2^ by Anton Paar in conjunction with the Oliver and Pharr method to evaluate the hardness and Young's modulus of the printed ethanol material. A square microstructure with dimensions of 140 × 140 × 5 µm^3^ was fabricated using a 7 kV acceleration voltage, an injection time of 175 s (corresponding to 840 nm per layer), and a total dose of 100 mC/cm^2^. The sample was mounted on a resin holder to ensure mechanical stability during testing. In the first testing sequence, force‐controlled progressive cyclic loading was applied from 0.2 to 10 mN over 50 cycles, with both loading and unloading rates set at 20 mN/min. Subsequently, depth‐controlled cyclic loading was performed on three different locations, ranging from 10 to 1200 nm penetration depth over 50 cycles, using the same loading/unloading rate. Finally, to probe shallow‐depth behavior and validate trends from progressive tests, constant‐depth multicycle indentations were performed at 80–100 nm and 250–300 nm, yielding repeated load–unload data with minimal substrate influence. We assumed a Poisson's ratio of 0.45 for the cross‐linked ethanol, based on typical values reported for similar polymeric materials such as PVA (0.38–0.49) [62].

We conducted a TEM analysis using a Titan E‐Cell 80–300ST system (FEI) and an acceleration voltage of 300 kV in an argon atmosphere. We subjected 3DIL grid patterns fabricated on the Si_3_N_4_ windows of wildfire chips by DENSolutions. We used in situ heating with a temperature ramp from 100°C to 1300°C and holding intervals at 900°C and 1100°C. The dwell time at the highest temperature was 6 h. To minimize sample alteration due to electron beam exposure, the sample was only exposed to the electron beam during image acquisition. Between image acquisitions, the electron beam was blanked.

FIB‐SEM experiments were performed with a Dual Beam FEI Helios nanoLab 600 system. Around 1 µm‐thick platinum was deposited to preserve the structure morphology. The Ga^+^ ion beam was set to 30 kV with a beam current of 0.46 nA to cut the patterns; afterward, SEM was performed with a through‐the‐lens detector to achieve the ideal imaging quality.

All the plotted data is processed using MATLAB R2021b. The figures are edited with Inkscape or Illustrator. The 3D‐generated figures are produced using Blender 4.1.

### 4.3 Culture of Endothelial Cells on 3DIL Fabricated Scaffolds

We printed 250 µm by 220 µm scaffolds on indium‐tin oxide (ITO) coated glass coverslips by Diamond Coatings, six honeycomb‐shaped and three log piles. The six honeycomb scaffolds had two layers of repeating hexagons. Half had hexagonal pores of 20 µm and half of 40 µm. The thickness of the hexagon sides was 10 µm for all designs. The three log pile scaffolds had pores of 20 µm and sides of 10 µm.

Immortalised HUVEC's (CI‐HUVECs; inscreenex) were cultured in HUVEC media (inscreenex; INS‐ME‐1011), supplemented with penicillin streptomycin (Gibco), according to the instructions of the supplier. Prior to seeding cells, the 3DIL fabricated substrates were sterilised by washing in 70% EtOH, carefully drying, and exposing the substrates to the bench UV light for 20 min. The substrates were placed in 6‐well plates and coated with a 0.5% gelatin solution, which was left to adsorb for 20 min in the incubator.

Cells were seeded at 10.000 cell/cm^2^ and cultured under normal conditions. 6 days post‐seeding, cells were fixed in 4% paraformaldehyde solution for 30 min at room temperature. The fixed cells were stained with DAPI and Phalloidin 555 (Invitrogen A30106) at 1× concentrations dissolved in PBS with 0.025% Triton X‐100. Samples were protected from light and incubated with the fluorescent stains for 1 h at room temperature, before washing 3 × 5 min with PBS.

Fluorescent images were acquired on a Nikon Eclipse Ti2 spinning disc confocal microscope equipped with an Andor iXon Ultra camera. Images were processed in Fiji.

### 4.4 Animal Surgery and Probe Implantation

We 3DIL patterned on platinum iridium metal electrodes (PI2PT30.01. A3; Microprobes for Life Sciences) profiled by WPI on one side, using ethanol and an acceleration voltage of 3 kV, an injection time of 40 s, and a dose of 30 mC/cm^2^. The design was lines of 2 µm by 2 µm circles with 1 µm spacing.

All surgical procedures were approved by the Danish National Committee on Health Research and conducted following the European Council's Convention for the Projection of Vertebrate Animals used for experimental and other scientific purposes, adhering to established animal research guidelines.

C57BL/6 mice (N = 4 mice) were anesthetized with 4% isoflurane for induction and maintained at 1%–1.5% during surgery. The body temperature was kept at 37°C throughout the procedure. Once reflexes were absent, a small skin incision was made to expose the skull. Using stereotaxic coordinates, burr holes were drilled at approximately −2 mm anterior‐posterior (AP) and ±1.5 mm medial‐lateral (ML) relative to bregma in both hemispheres.

Before the probe insertion, probe tips were immersed in the BioTracker 490 (SCT106, Sigma) to enable visualization of implantation sites via green fluorescence. The probes were then carefully lowered into the cortex by hand – a control probe in the left hemisphere and a cross‐linked ethanol‐coated probe in the right. Burr holes were sealed with Kwik‐Sil (World Precision Instrument, USA), a biocompatible silicone adhesive suitable for chronic implantation. After allowing the sealant to set, the scalp incision was closed with silk sutures.

Two weeks post‐implantation, mice were transcardially perfused with 4% paraformaldehyde (PFA). The probes from both hemispheres were carefully removed, and brains were extracted and post‐fixed in 4% PFA at 4°C for 2 h. Brains were then transferred to 30% sucrose solution at 4°C for 48 h, followed by rapid freezing in dry ice‐chilled isopentane. Samples were stored at ∼80°C until use. Cellular and molecular responses surrounding the probe‐implanted sites were compared by IHC analysis.

Brains were sectioned by cryostat into coronal 50‐µm‐thick slices. Sections were rinsed in phosphate‐buffered saline (PBS) for 3 times, then blocked overnight at 4°C in 1% bovine serum albumin with 0.5% trition‐X. Sections were further incubated at 4°C for two nights with primary antibody: mouse anti‐NeuN antibody (1:500, 94403, CST), mouse anti‐GFAP antibody (1:1000, MAB360, EMD Millipore), rabbit anti‐NeuN antibody (1:100–1:300, ab177487, Abcam), and rabbit anti‐Iba1 antibody (1:100, SAB5701363, Sigma). After washing in PBS 3 times. sections were incubated overnight at 4°C with secondary antibodies: donkey anti‐rabbit IgG H&L (Alexa Fluor 568) preadsorbed antibody (1:500, ab175692, Abcam) and donkey anti‐mouse IgG H&L (Alexa Fluor 647) antibody (1:500, ab150107, Abcam). Sections were then stained with Hoechst 33342 (1:6,000, B2261, Sigma) for 7 min and mounted using SlowFade Diamond antifade mountant (Invitrogen, S36963). Stained sections were imaged using Zeiss Axioscan 7 with a 20× objective. Images were captured at a resolution of approximately 0.172 µm/pixel (x and y) and 3 µm (z‐step) in coronal brain slices around the implant sites.

### 4.5 Data Analysis and Statistics

Glial reactivity surrounding probe implants was assessed by semi‐quantitative immunofluorescence analysis of GFAP and Iba1 as a function of distance from the probe tract. For each implant, 2–5 coronal brain sections at matched anatomical levels were analyzed. Section thickness, antibody batches, staining protocols, and imaging parameters were kept identical across experimental groups. All images were acquired using the same objective, spatial resolution, and exposure or laser settings within each staining batch, and files were renamed for blinded analysis.

The probe tract was identified using BioTracker 490 fluorescence, and the void itself was excluded from all measurements. Concentric distance bins centered on the probe tract (0–50, 50–100, and 100–150 µm, Figure S5) were generated and applied consistently across all images.

GFAP and Iba1 signals surrounding the probe tract were analyzed independently using a single predefined global threshold applied uniformly across the dataset. For each distance bin, the threshold‐positive area and total bin area were measured to calculate the percentage of area positive.

For sham controls, a 688.27 µm × 688.27 µm cortical region located 1000 µm away from the probe tract was selected. The percentage of marker‐positive area was calculated as the ratio of threshold‐positive area to total region area. A paired *t*‐test was performed to assess statistical significance across ROIs between the control and cross‐linked ethanol‐coated probe.

## Conflicts of Interest

A.H. is a co‐founder of Copenhagen Microsystems (Founded December 2025).

## Supporting information




**Supporting File**: advs75341‐sup‐0001‐SuppMat.docx.

## Data Availability

The data that support the findings of this study are available from the corresponding author upon reasonable request.
