# Supplementary material for: Solid Ethanol as a Renewable, Low‐Toxicity, Electron‐Beam Direct Write, and Biomedical Material
Source: Adv Sci (Weinh). 2026 Apr 27;13(38):e75341. doi: 10.1002/advs.75341 (PMC13335537; doi:10.1002/advs.75341)
Supplement: Supplementary file 1 — Supporting File: advs75341‐sup‐0001‐SuppMat.docx. [file ADVS-13-e75341-s001.docx]

Supplementary Information

**Solid Ethanol as a Renewable, Low-Toxicity, Electron-Beam Direct Write, and Biomedical Material**

Bruno Perdigão^1, 4^, Bingdong Chang^1^, Lechan Tao^2^, Kayeon Kim^2^, Anne Zebitz Eriksen^3^, Gwendoline A. E. Anand^1^, Malte Alexander Schönhoff^1^, Joachim Lyngholm-Kjærby^1^, Guilherme Ferreira^1^, Xiyuan Liu^1^, Su Genelioglu^1^, Neha Zahoor^5^, Johan Ulrik Lind^3^, Alice Bastos da Silva Fanta^5^, Thomas Willum Hansen^5^, Changsi Cai^2*^, Anpan Han^1*^


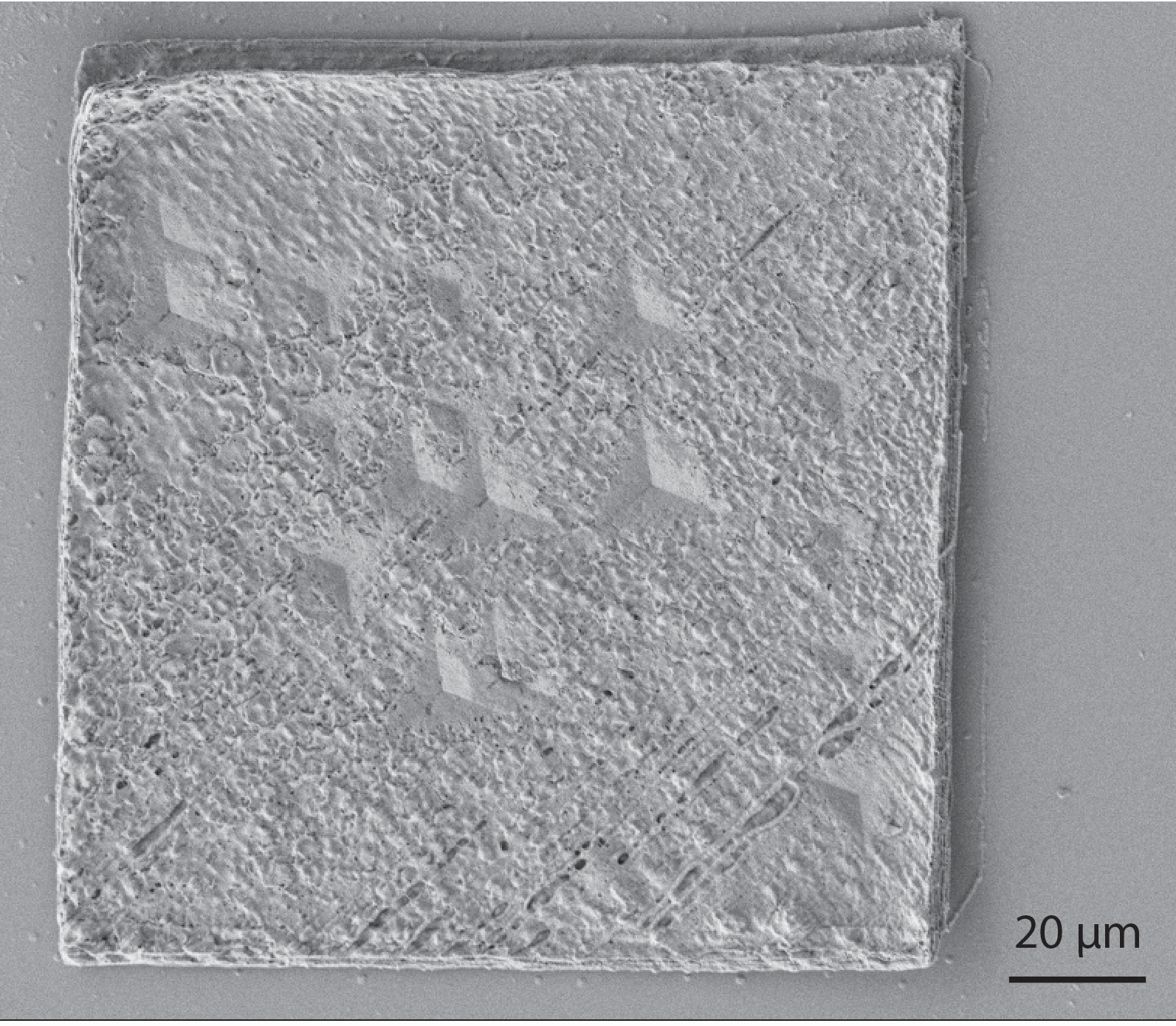


**Figure S1.** SEM image showing the 3DIL-processed ethanol ice structure for nanoindentation test. The indentation sites can be clearly observed, which are distributed across the surface.


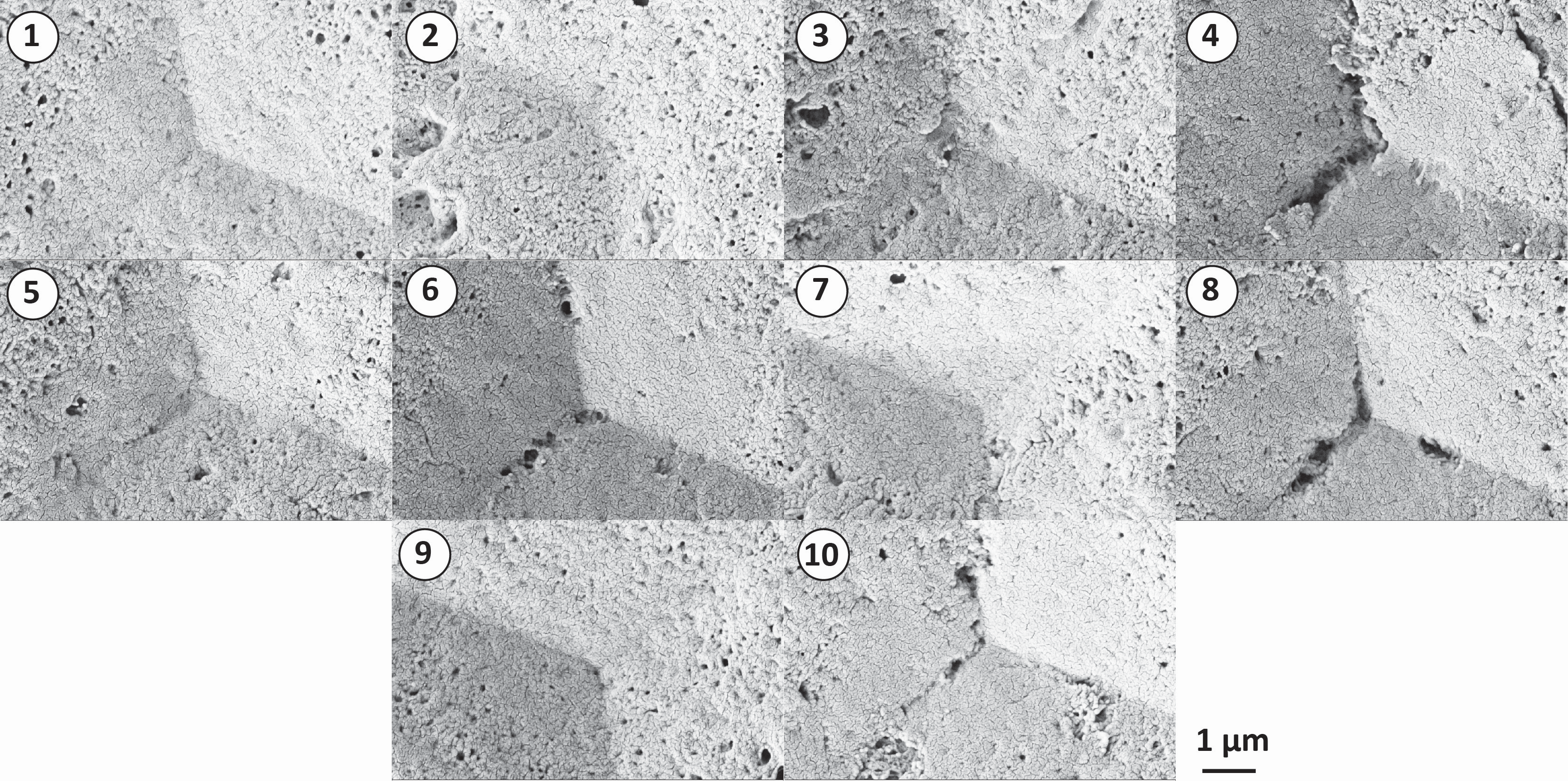


**Figure S2.** SEM images showing the morphology of 10 different nanoindentation sites. The surface porosity can be observed directly.


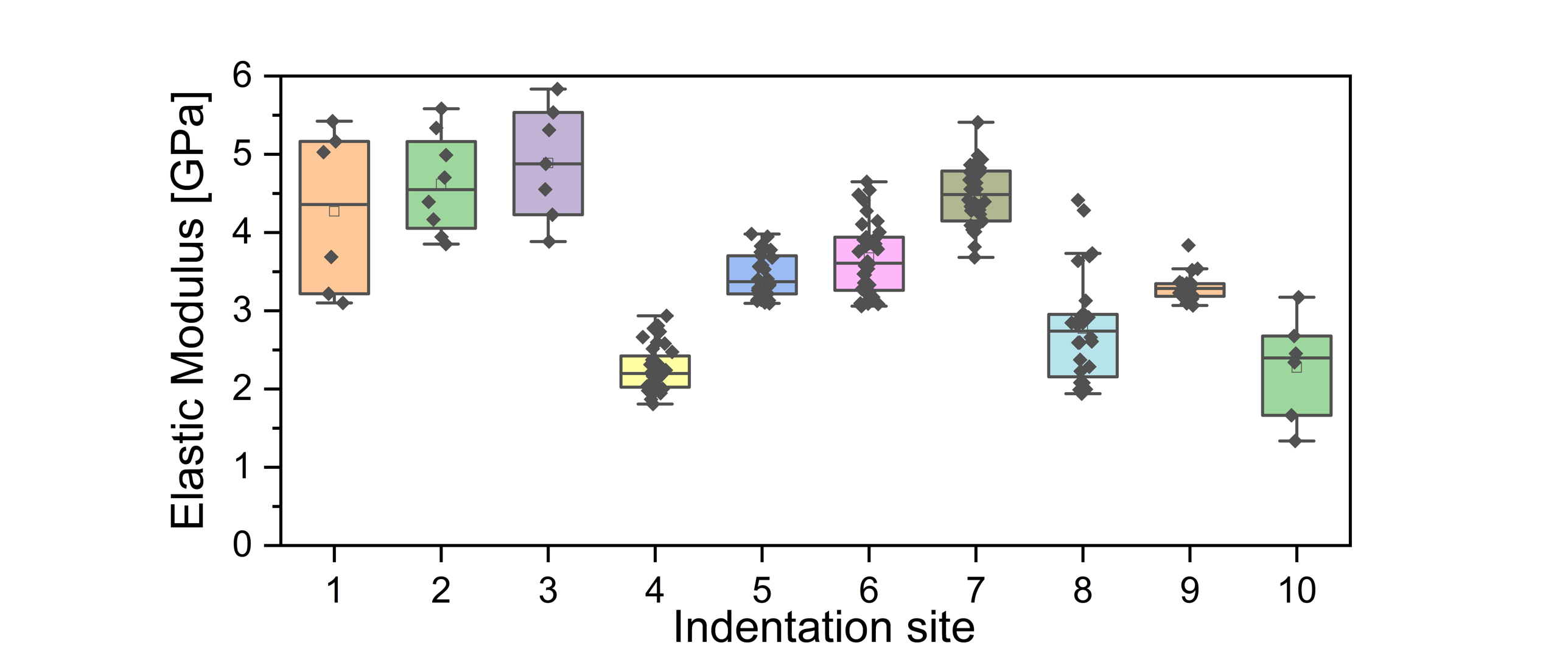


**Figure S3.** Statistics of nanoindentation measurements from 10 different indentation sites.

**Figure S4**. HUVEC cell density on prints and control tissue culture plastic after 6 days of culture. Bar shows the group mean and the error bars show the standard deviation (SD). We performed a Welch two sample t-test on the data to compare the means. T=1.67, df =8.197, p= 0.127 meaning no significant difference between the two means. Statistics were performed using R.


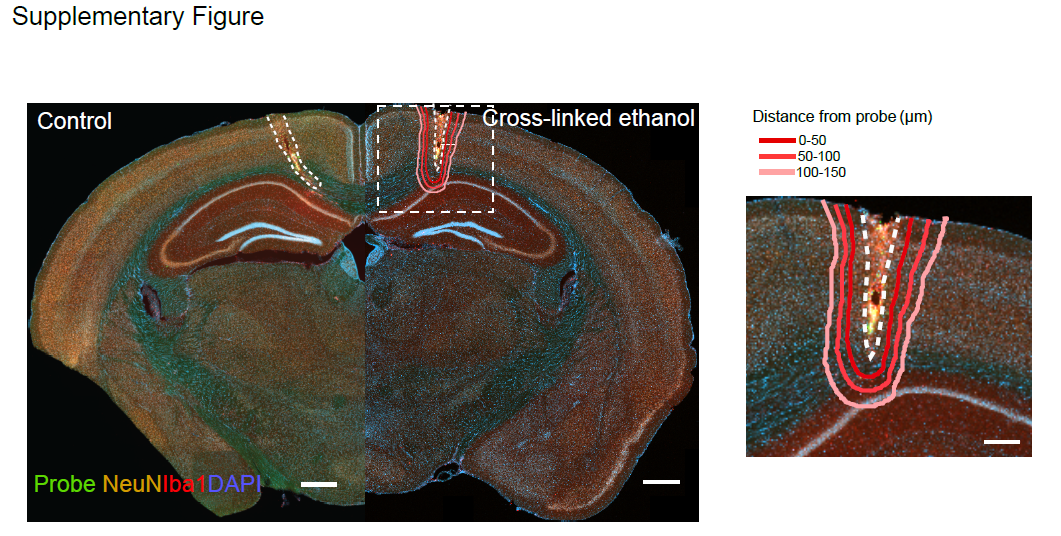


**Figure S5**. (Left): Same as Fig.6(c), but contours delineate the regions used for quantification of glial reactivity. Scale bar: 500 μm. (Right): Magnified view of the left panel showing distance bins from the probe tract for statistical analysis. Scale bar: 150 μm.
